# Inferring the effectiveness of government interventions against COVID-19

**DOI:** 10.1126/science.abd9338

**Published:** 2020-12-15

**Authors:** Jan M. Brauner, Sören Mindermann, Mrinank Sharma, David Johnston, John Salvatier, Tomáš Gavenčiak, Anna B. Stephenson, Gavin Leech, George Altman, Vladimir Mikulik, Alexander John Norman, Joshua Teperowski Monrad, Tamay Besiroglu, Hong Ge, Meghan A. Hartwick, Yee Whye Teh, Leonid Chindelevitch, Yarin Gal, Jan Kulveit

**Affiliations:** 1 Oxford Applied and Theoretical Machine Learning (OATML) Group, Department of Computer Science, University of Oxford, Oxford, UK.; 2 Future of Humanity Institute, University of Oxford, Oxford, UK.; 3 Department of Statistics, University of Oxford, Oxford, UK.; 4 Department of Engineering Science, University of Oxford, Oxford, UK.; 5 College of Engineering and Computer Science, Australian National University, Canberra, Australia.; 6 Quantified Uncertainty Research Institute, San Francisco, CA, USA.; 7 Independent scholar, Prague, Czech Republic.; 8 Harvard John A. Paulson School of Engineering and Applied Sciences, Harvard University, Cambridge, MA, USA.; 9 School of Computer Science, University of Bristol, Bristol, UK.; 10 School of Medical Sciences, University of Manchester, Manchester, UK.; 11 Independent scholar, London, UK.; 12 Mathematical, Physical and Life Sciences (MPLS) Doctoral Training Centre, University of Oxford, Oxford, UK.; 13 Faculty of Public Health and Policy, London School of Hygiene and Tropical Medicine, London, UK.; 14 Department of Health Policy, London School of Economics and Political Science, London, UK.; 15 Faculty of Economics, University of Cambridge, Cambridge, UK.; 16 Engineering Department, University of Cambridge, Cambridge, UK.; 17 Tufts Initiative for the Forecasting and Modeling of Infectious Diseases, Tufts University, Boston, MA, USA.; 18 Medical Research Council (MRC) Centre for Global Infectious Disease Analysis, School of Public Health, Imperial College London, London, UK.; 19 Abdul Latif Jameel Institute for Disease and Emergency Analytics (J-IDEA), School of Public Health, Imperial College London, London, UK.

## Abstract

Governments are attempting to control the COVID-19 pandemic with nonpharmaceutical interventions (NPIs). However, the effectiveness of different NPIs at reducing transmission is poorly understood. We gathered chronological data on the implementation of NPIs for several European and non-European countries between January and the end of May 2020. We estimated the effectiveness of these NPIs, which range from limiting gathering sizes and closing businesses or educational institutions to stay-at-home orders. To do so, we used a Bayesian hierarchical model that links NPI implementation dates to national case and death counts and supported the results with extensive empirical validation. Closing all educational institutions, limiting gatherings to 10 people or less, and closing face-to-face businesses each reduced transmission considerably. The additional effect of stay-at-home orders was comparatively small.

## How to hold down transmission

Early in 2020, severe acute respiratory syndrome coronavirus 2 (SARS-CoV-2) transmission was curbed in many countries by imposing combinations of nonpharmaceutical interventions. Sufficient data on transmission have now accumulated to discern the effectiveness of individual interventions. Brauner *et al.* amassed and curated data from 41 countries as input to a model to identify the individual nonpharmaceutical interventions that were the most effective at curtailing transmission during the early pandemic. Limiting gatherings to fewer than 10 people, closing high-exposure businesses, and closing schools and universities were each more effective than stay-at-home orders, which were of modest effect in slowing transmission.

*Science*, this issue p. eabd9338

## Abstract (structured)

**INTRODUCTION:**

Governments across the world have implemented a wide range of nonpharmaceutical interventions (NPIs) to mitigate the spread of severe acute respiratory syndrome coronavirus 2 (SARS-CoV-2). Given the increasing death toll of the pandemic and the social cost of some interventions, it is critical to understand their relative effectiveness. By considering the effects that interventions had on transmission during the first wave of the outbreak, governments can make more-informed decisions about how to control the pandemic.

**RATIONALE:**

Rigorously studying the effectiveness of individual interventions poses considerable methodological challenges. Simulation studies can explore scenarios, but they make strong assumptions that may be difficult to validate. Data-driven, cross-country modeling comparing the timing of national interventions to the subsequent numbers of cases or deaths is a promising alternative approach. We have collected chronological data on the implementation of several interventions in 41 countries between January and the end of May 2020, using independent double entry by researchers to ensure high data quality. Because countries deployed different combinations of interventions in different orders and with different outcomes, it is possible to disentangle the effect of individual interventions. We estimate the effectiveness of specific interventions with a Bayesian hierarchical model by linking intervention implementation dates to national case and death counts. We partially pool NPI effectiveness to allow for country-specific NPI effects. Our model also accounts for uncertainty in key epidemiological parameters, such as the average delay from infection to death. However, intervention effectiveness estimates should only be used for policy-making if they are robust across a range of modeling choices. We therefore support the results with extensive empirical validation, including 11 sensitivity analyses under 206 experimental conditions. In these analyses, we show how results change when we vary the data, the epidemiological parameters, or the model structure or when we account for confounders.

**RESULTS:**

While exact intervention effectiveness estimates varied with modeling assumptions, broader trends in the results were highly consistent across experimental conditions. To describe these trends, we categorized intervention effect sizes as small, moderate, or large, corresponding to posterior median reductions in the reproduction number *R* of <17.5%, between 17.5 and 35%, and >35%, respectively. Across all experimental conditions, all interventions could robustly be placed in one or two of these categories. Closing both schools and universities was consistently highly effective at reducing transmission at the advent of the pandemic. Banning gatherings was effective, with a large effect size for limiting gatherings to 10 people or less, a moderate-to-large effect for 100 people or less, and a small-to-moderate effect for 1000 people or less. Targeted closures of face-to-face businesses with a high risk of infection, such as restaurants, bars, and nightclubs, had a small-to-moderate effect. Closing most nonessential businesses delivering personal services was only somewhat more effective (moderate effect). When these interventions were already in place, issuing a stay-at-home order had only a small additional effect. These results indicate that, by using effective interventions, some countries could control the epidemic while avoiding stay-at-home orders.

**CONCLUSION:**

We estimated the effects of nonpharmaceutical interventions on COVID-19 transmission in 41 countries during the first wave of the pandemic. Some interventions were robustly more effective than others. This work may provide insights into which areas of public life require additional interventions to be able to maintain activity despite the pandemic. However, because of the limitations inherent in observational study designs, our estimates should not be seen as final but rather as a contribution to a diverse body of evidence, alongside other retrospective studies, simulation studies, and experimental trials.

## Teaser

The effect of nonpharmaceutical interventions on SARS-CoV-2 transmission during the early phase of the pandemic is quantified.

## Graphical abstract

**Figure:**
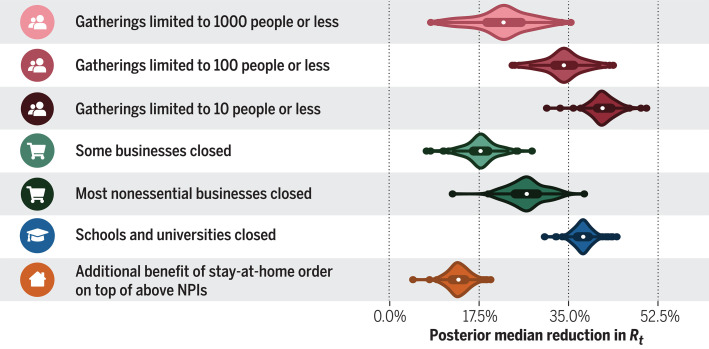
Median intervention effectiveness estimates across a suite of 206 analyses with different epidemiological parameters, data, and modeling assumptions. Bayesian inference using a semimechanistic hierarchical model with observed national case and death data across 41 countries between January and May 2020 is used to infer the effectiveness of several nonpharmaceutical interventions. Although precise effectiveness estimates depend on the assumed data and parameters, there are clear trends across the experimental conditions. Violins show kernel density estimates of the posterior median effectiveness across the sensitivity analysis. *R_t_*, instantaneous reproduction number.

Worldwide, governments have mobilized resources to fight the COVID-19 pandemic. A wide range of nonpharmaceutical interventions (NPIs) has been deployed, including stay-at-home orders and the closure of all nonessential businesses. Recent analyses show that these large-scale NPIs were jointly effective at reducing the virus’s effective reproduction number *R_t_* (*1*), but it is still largely unknown how effective individual NPIs were. As more data become available, we can move beyond estimating the combined effect of a bundle of NPIs and begin to understand the effects of individual interventions. This can help governments efficiently control the epidemic, by focusing on the most effective NPIs to ease the burden put on the population.

A promising way to estimate NPI effectiveness is data-driven, cross-country modeling: inferring effectiveness by relating the NPIs implemented in different countries to the course of the epidemic in these countries. To disentangle the effects of individual NPIs, we need to leverage data from multiple countries with diverse sets of interventions in place. Previous data-driven studies (table S8) estimate effectiveness for individual countries (*2*–*4*) or NPIs, although some exceptions do exist [(*1*, *5*–*8*); summarized in table S7]. In contrast, we evaluated the impact of several NPIs on the epidemic’s growth in 34 European and 7 non-European countries. If all countries implemented the same set of NPIs on the same day, the individual effect of each NPI would be unidentifiable. However, the COVID-19 response was far less coordinated: Countries implemented different sets of NPIs at different times and in different orders (Fig. 1).

**Fig. 1:**
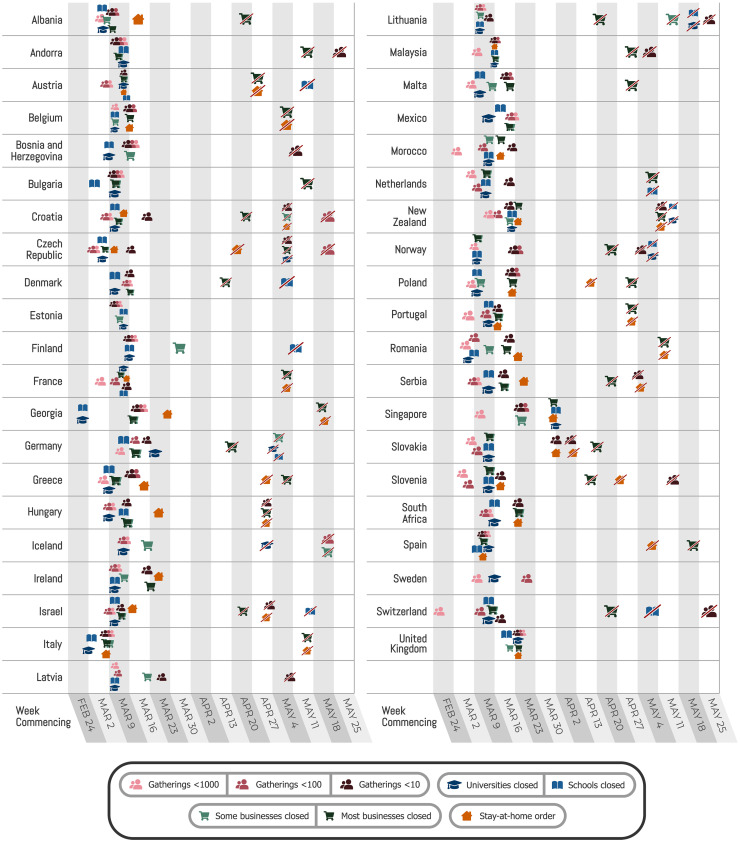
Timing of NPI implementations in early 2020. Crossed-out icons signify when an NPI was lifted. Detailed definitions of the NPIs are given in Table 1.

Even with diverse data from many countries, estimating NPI effects remains a challenging task. To begin with, models are based on uncertain epidemiological parameters; our NPI effectiveness study incorporates some of this uncertainty directly into the model. Furthermore, the data are retrospective and observational, meaning that unobserved factors could confound the results. Also, NPI effectiveness estimates can be highly sensitive to arbitrary modeling decisions, as shown by two recent replication studies (*9*, *10*). And finally, large-scale public NPI datasets suffer from frequent inconsistencies (*11*) and missing data (*12*). Hence, the data and the model must be carefully validated if they are to be used to guide policy decisions. We have collected a large public dataset on NPI implementation dates that has been validated by independent double entry, and we have extensively validated our effectiveness estimates. This validation of data and model is a crucial but often absent or incomplete element of COVID-19 NPI effectiveness studies (*10*).

Our results provide insight on the amount of COVID-19 transmission associated with various areas and activities of public life, such as gatherings of different sizes. Therefore, they may inform the packages of interventions that countries implement to control transmission in current and future waves of infections. However, we need to be careful when interpreting this study’s results. We only analyzed the effect NPIs had between January and the end of May 2020, and NPI effectiveness may change over time as circumstances change. Lifting an NPI does not imply that transmission will return to its original level, and our window of analysis does not include relaxation of NPIs. These and other limitations are detailed in the Discussion section.

## Cross-country NPI effectiveness modeling

We analyzed the effects of seven commonly used NPIs between 22 January and 30 May 2020. All NPIs aimed to reduce the number of contacts within the population (Table 1). If a country lifted an NPI before 30 May, the window of analysis for that country terminates on the day of the lifting (see Materials and methods). To ensure high data quality, all NPI data were independently entered by two of the authors (independent double entry) using primary sources and then manually compared with several public datasets. Data on confirmed COVID-19 cases and deaths were taken from the Johns Hopkins Center for Systems Science and Engineering (CSSE) COVID-19 Dataset (*13*). The data used in this study, including sources, are available online (*14*).

**Table 1:** NPIs included in the study.

**NPI**	**Description**
**Gatherings limited to** **1000 people or less**	A country has set a size limit on gatherings. The limit is at most 1000 people often less), and gatherings above the maximum size are disallowed. For example, a ban on gatherings of 500 people or more would be classified as “gatherings limited to 1000 or less,” but a ban on gatherings of 2000 people or more would not.
**Gatherings limited to** **100 people or less**	A country has set a size limit on gatherings. The limit is at most 100 people (often less).
**Gatherings limited to** **10 people or less**	A country has set a size limit on gatherings. The limit is at most 10 people (often less).
**Some businesses closed**	A country has specified a few kinds of face-to-face businesses that are considered high risk and need to suspend operations (blacklist). Common examples are restaurants, bars, nightclubs, cinemas, and gyms. By default, businesses are not suspended.
**Most nonessential** **businesses closed**	A country has suspended the operations of many face-to-face businesses. By default, face-to-face businesses are suspended unless they are designated as essential (whitelist).
**Schools closed**	A country has closed most or all schools.
**Universities closed**	A country has closed most or all universities and higher-education facilities.
**Stay-at-home order**	An order for the general public to stay at home has been issued. This is mandatory, not just a recommendation. Exemptions are usually granted for certain purposes (such as shopping, exercise, or going to work) or, more rarely, for certain times of the day. Whenever countries in our dataset introduced stay-at-home orders, they essentially always also implemented, or already had in place, all other NPIs listed in this table. All these are encoded as distinct NPIs in the data. In our results, we thus estimate the additional effect of a stay-at-home order on top of all other NPIs.

We estimated the effectiveness of NPIs with a Bayesian hierarchical model. We used case and death data from each country to infer the number of new infections at each point in time, which is itself used to infer the (instantaneous) reproduction number *R_t_* over time. NPI effects were then estimated by relating the daily reproduction numbers to the active NPIs, across all days and countries. This relatively simple, data-driven approach allowed us to sidestep assumptions about contact patterns and intensity, infectiousness of different age groups, and so forth that are typically required in modeling studies. This approach also allowed us to directly model many sources of uncertainty, such as uncertain epidemiological parameters, differences in NPI effectiveness between countries, unknown changes in testing and infection fatality rates, and the effect of unobserved influences on *R_t_*. The code is available online (*14*).

## Effectiveness of individual NPIs

**Fig. 2:**
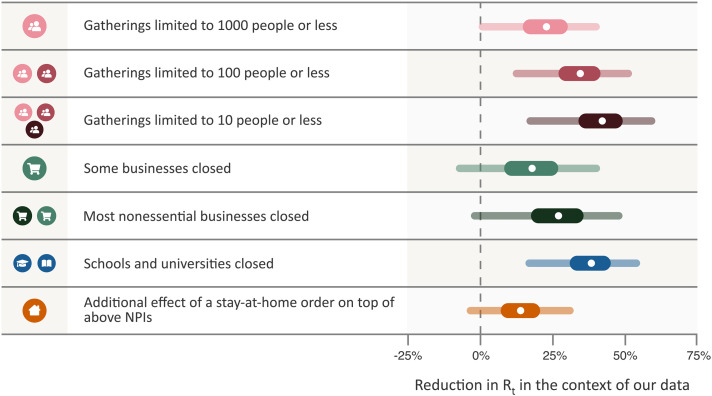
NPI effectiveness under default model settings. Posterior percentage reductions in *R_t_* with median, 50%, and 95% prediction intervals shown. Prediction intervals reflect many sources of uncertainty, including NPI effectiveness varying by country and uncertainty in epidemiological parameters. A negative 1% reduction refers to a 1% increase in *R_t_*. “Schools and universities closed” shows the joint effect of closing both schools and universities; the individual effect of closing just one will be smaller (see text). Cumulative effects are shown for hierarchical NPIs (gathering bans and business closures), that is, the result for “Most nonessential businesses closed” shows the cumulative effect of two NPIs with separate parameters and icons—closing some (high-risk) businesses, and additionally closing most remaining (non-high-risk but nonessential) businesses given that some businesses are already closed.

Our model enabled us to estimate the individual effectiveness of each NPI, expressed as a percentage reduction in *R_t_*. We quantified uncertainty with Bayesian prediction intervals, which are wider than standard credible intervals. Bayesian prediction intervals reflect differences in NPI effectiveness across countries among several other sources of uncertainty. They are analogous to the standard deviation of the effectiveness across countries rather than the standard error of the mean effectiveness. Under the default model settings, the percentage reduction in *R_t_* (with 95% prediction interval; Fig. 2) associated with each NPI was as follows: limiting gatherings to 1000 people or less: 23% (0 to 40%); limiting gatherings to 100 people or less: 34% (12 to 52%); limiting gatherings to 10 people or less: 42% (17 to 60%); closing some high-risk face-to-face businesses: 18% (−8 to 40%); closing most nonessential face-to-face businesses: 27% (−3 to 49%); closing both schools and universities in conjunction: 38% (16 to 54%); and issuing stay-at-home orders (additional effect on top of all other NPIs): 13% (−5 to 31%). Note that we were not able to robustly disentangle the individual effects of closing only schools or only universities, because these NPIs were implemented on the same day or in close succession in most countries [except Iceland and Sweden, where only universities were closed (see also fig. S21)]. We thus reported “schools and universities closed” as one NPI.

Some NPIs frequently co-occurred, i.e., were partly collinear. However, we were able to isolate the effects of individual NPIs, because the collinearity was imperfect and our dataset large. For every pair of NPIs, we observed one without the other for 504 days across all countries (country-days) on average (table S5). The minimum number of country-days for any NPI pair is 148 (for limiting gatherings to 1000 or 100 attendees). Additionally, under excessive collinearity, and insufficient data to overcome it, individual effectiveness estimates would be highly sensitive to variations in the data and model parameters (*15*). Indeed, high sensitivity prevented Flaxman *et al*. (*1*), who had a smaller dataset, from disentangling NPI effects (*9*). In contrast, our effectiveness estimates are substantially less sensitive (see below). Finally, the posterior correlations between the effectiveness estimates are weak, further suggesting manageable collinearity (fig. S22).

## Effectiveness of NPI combinations

**Fig. 3:**
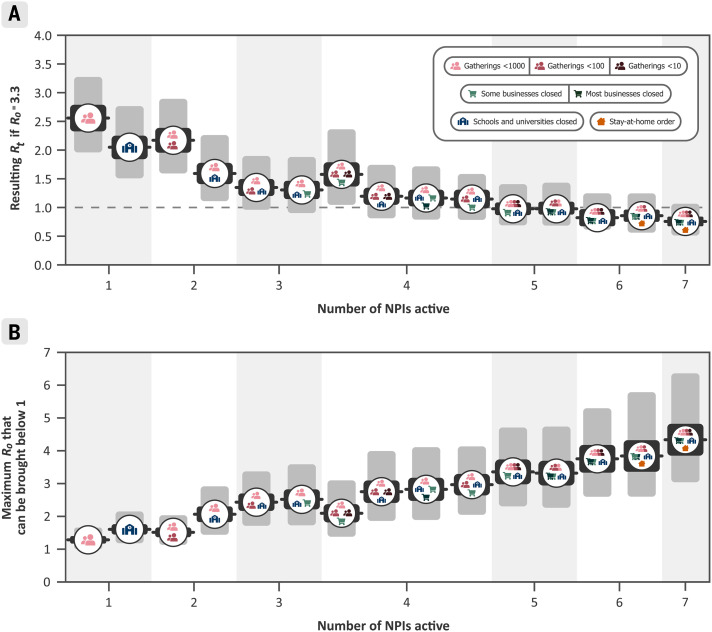
Combined NPI effectiveness for the 15 most commonly implemented sets of NPIs in our data. Black and gray bars denote 50% and 95% Bayesian prediction intervals, respectively. (**A**) Predicted *R_t_* after implementation of each set of NPIs, assuming *R*_0_ = 3.3. (**B**) Maximum *R*_0_ that can be reduced to *R_t_* below 1 by common sets of NPIs. Readers can interactively explore the effects of all sets of NPIs, while setting *R*_0_ and adjusting NPI effectiveness to local circumstances, with our online mitigation calculator (*16*).

Although the correlations between the individual estimates were weak, we took them into account when evaluating combined NPI effectiveness. For example, if two NPIs frequently co-occur, there may be more certainty about the combined effectiveness than about the effectiveness of each NPI individually. Figure 3 shows the combined effectiveness of the sets of NPIs that are most common in our data. In combination, the NPIs in this study reduced *R_t_* by 77% (67 to 85%). Across countries, the mean *R_t_* without any NPIs (i.e., the *R*_0_) was 3.3 (table S4). Starting from this number, the estimated *R_t_* likely could have been brought below 1 by closing schools and universities, closing high-risk businesses, and limiting gathering sizes to at most 10 people. Readers can interactively explore the effects of sets of NPIs with our online mitigation calculator (*16*). A comma-separated value file containing the joint effectiveness of all NPI combinations is available online (*14*).

## Sensitivity and validation

**Fig. 4:**
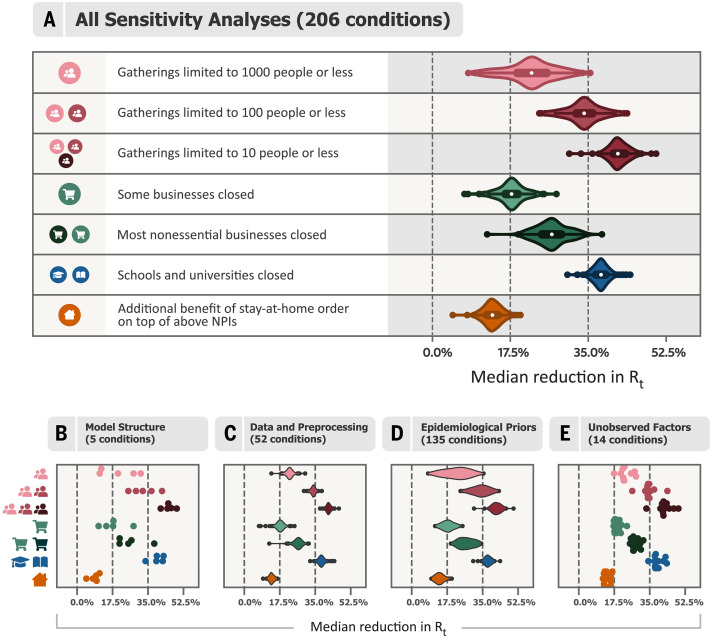
Median NPI effectiveness across the sensitivity analyses. (**A**) Median NPI effectiveness (reduction in *R_t_*) when varying different components of the model or the data in 206 experimental conditions. Results are displayed as violin plots, using kernel density estimation to create the distributions. Inside the violins, the box plots show median and interquartile range. The vertical lines mark 0, 17.5, and 35% (see text). (**B** to **E**) Categorized sensitivity analyses. (B) Sensitivity to model structure. Using only cases or only deaths as observations (two experimental conditions; fig. S7); varying the model structure (three conditions; fig. S8, left). (C) Sensitivity to data and preprocessing. Leaving out countries from the dataset (42 conditions; figs. S5 and S21); varying the threshold below which cases and deaths are masked (eight conditions; fig. S13); sensitivity to correcting for undocumented cases and to country-level differences in case ascertainment (two conditions; fig. S6). (D) Sensitivity to epidemiological parameters. Jointly varying the means of the priors over the means of the generation interval, the infection-to-case-confirmation delay, and the infection-to-death delay (125 conditions; fig. S10); varying the prior over *R*_0_ (four conditions; fig. S11); varying the prior over NPI effect parameters (three conditions; fig. S11); varying the prior over the degree to which NPI effects vary across countries (three conditions; fig. S12). (E) Sensitivity to unobserved factors influencing *R_t_*. Excluding observed NPIs one at a time (eight conditions; fig. S9); controlling for additional NPIs from a different dataset (six conditions; fig. S9).

We performed a range of validation and sensitivity experiments (figs. S2 to S19). First, we analyzed how the model extrapolated to countries that did not contribute data for fitting the model, and we found that it could generate calibrated forecasts for up to 2 months, with uncertainty increasing over time. Multiple sensitivity analyses showed how the results changed when we modified the priors over epidemiological parameters, excluded countries from the dataset, used only deaths or confirmed cases as observations, varied the data preprocessing, and more. Finally, we tested our key assumptions by showing results for several alternative models [structural sensitivity (*10*)] and examined possible confounding of our estimates by unobserved factors influencing *R_t_*. In total, we considered NPI effectiveness under 206 alternative experimental conditions (Fig. 4A). Compared with the results obtained under our default settings (Figs. 2 and 3), median NPI effectiveness varied under alternative plausible experimental conditions. However, the trends in the results are robust, and some NPIs outperformed others under all tested conditions. Although we tested large ranges of plausible values, our experiments did not include every possible source of uncertainty.

We categorized NPI effects into small, moderate, and large, which we define as a posterior median reduction in *R_t_* of <17.5%, between 17.5 and 35%, and >35%, respectively (vertical lines in Fig. 4). Four of the NPIs fell into the same category across a large fraction of experimental conditions: closing both schools and universities was associated with a large effect in 96% of experimental conditions, and limiting gatherings to 10 people or less had a large effect in 99% of conditions. Closing most nonessential businesses had a moderate effect in 98% of conditions. Issuing stay-at-home orders (that is, in addition to the other NPIs) fell into the “small effect” category in 96% of experimental conditions. Three NPIs fell less clearly into one category: Limiting gatherings to 1000 people or less had a small-to-moderate effect (moderate in 81% of conditions) while limiting gatherings to 100 people or less had a moderate-to-large effect (moderate in 66% of conditions). Finally, closing some high-risk businesses, including bars, restaurants, and nightclubs, had a small-to-moderate effect (moderate in 58% of conditions). Limiting gatherings to 1000 people or less was the NPI with the highest variation in median effectiveness across the experimental conditions (Fig. 4A), which may reflect this NPI’s partial collinearity with limiting gatherings to 100 people or less.

Aggregating all sensitivity analyses can hide sensitivity to specific assumptions. We display the median NPI effects in four categories of sensitivity analyses (Fig. 4, B to E), and each individual sensitivity analysis is shown in the supplementary materials. The trends in the results are also stable within these categories.

## Discussion

We used a data-driven approach to estimate the effects that seven nonpharmaceutical interventions had on COVID-19 transmission in 41 countries between January and the end of May 2020. We found that several NPIs were associated with a clear reduction in *R_t_*, in line with mounting evidence that NPIs are effective at mitigating and suppressing outbreaks of COVID-19. Furthermore, our results indicate that some NPIs outperformed others. While the exact effectiveness estimates vary with modeling assumptions, the broad conclusions discussed below are largely robust across 206 experimental conditions in 11 sensitivity analyses.

Business closures and gathering bans both seem to have been effective at reducing COVID-19 transmission. Closing most nonessential face-to-face businesses was only somewhat more effective than targeted closures, which only affected businesses with high infection risk, such as bars, restaurants, and nightclubs (see also Table 1). Therefore, targeted business closures can be a promising policy option in some circumstances. Limiting gatherings to 10 people or less was more effective than limits of up to 100 or 1000 people and had a more robust effect estimate. Note that our estimates are derived from data between January and May 2020, a period when most gatherings were likely indoors owing to the weather.

Whenever countries in our dataset introduced stay-at-home orders, they essentially always also implemented, or already had in place, all other NPIs in this study. We accounted for these other NPIs separately and isolated the effect of ordering the population to stay at home, in addition to the effect of all other NPIs. In accordance with other studies that took this approach (*2*, *6*), we found that issuing a stay-at-home order had a small effect when a country had already closed educational institutions and nonessential businesses and had banned gatherings. In contrast, Flaxman *et al*. (*1*) and Hsiang *et al*. (*3*) included the effect of several NPIs in the effectiveness of their stay-at-home order (or “lockdown”) NPIs and accordingly found a large effect for this NPI. Our finding suggests that some countries may have been able to reduce *R_t_* to <1 without a stay-at-home order (Fig. 3) by issuing other NPIs.

We found a large effect for closing both schools and universities in conjunction, which was remarkably robust across different model structures, variations in the data, and epidemiological assumptions (Fig. 4). This effect remained robust when controlling for NPIs excluded from our study (fig. S9). Our approach cannot distinguish direct effects on transmission in schools and universities from indirect effects, such as the general population behaving more cautiously after school closures signaled the gravity of the pandemic. Additionally, because school and university closures were implemented on the same day or in close succession in most of the countries we studied, our approach cannot distinguish their individual effects (fig. S21). This limitation likely also holds for other observational studies that do not include data on university closures and estimate only the effect of school closures (*1*–*3*, *5*–*8*). Furthermore, our study does not provide evidence on the effect of closing preschools and nurseries.

Previous evidence on the role of pupils and students in transmission is mixed. Although infected young people (~12 to 25 years of age) are often asymptomatic, they appear to shed similar amounts of virus as older people (*17*, *18*) and might therefore infect higher-risk individuals. Early data suggested that children and young adults had a notably lower observed incidence rate than older adults—whether this was due to school and university closures remains unknown (*19*–*22*). In contrast, the recent resurgence of cases in European countries has been concentrated in the age group corresponding to secondary school and higher education (especially the latter) and is now spreading to older age groups as well as primary school–aged children (*23*, *24*). Primary schools may be generally less affected than secondary schools (*20*, *25*–*28*), perhaps partly because children under the age of 12 are less susceptible to SARS-CoV-2 (*29*).

Our study has several limitations. (i) NPI effectiveness may depend on the context of implementation, such as the presence of other NPIs, country demographics, and specific implementation details. Our results thus need to be interpreted as indicating the effectiveness in the contexts in which the NPI was implemented in our data (*10*). For example, in a country with a comparatively old population, the effectiveness of closing schools and universities would likely have been on the lower end of our prediction interval. Expert judgment should thus be used to adjust our estimates to local circumstances. (ii) *R_t_* may have been reduced by unobserved NPIs or voluntary behavior changes such as mask-wearing. To investigate whether the effect of these potential confounders could be falsely attributed to the observed NPIs, we performed several additional analyses and found that our results are stable to a range of unobserved factors (fig. S9). However, this sensitivity check cannot provide certainty, and investigating the role of unobserved factors is an important topic to explore further. (iii) Our results cannot be used without qualification to predict the effect of lifting NPIs. For example, closing schools and universities in conjunction seems to have greatly reduced transmission, but this does not mean that reopening them will necessarily cause infections to soar. Educational institutions can implement safety measures, such as reduced class sizes, as they reopen. However, the nearly 40,000 confirmed cases associated with universities in the United Kingdom since they reopened in September 2020 show that educational institutions may still play a large role in transmission, despite safety measures (*30*). (iv) We do not have data on some promising interventions, such as testing, tracing, and case isolation. These interventions could become an important part of a cost-effective epidemic response (*31*), but we did not include them because it is difficult to obtain comprehensive data on their implementation. In addition, although the data are more readily available, it is difficult to estimate the effect of mask-wearing in public spaces because there was limited public life as a result of other NPIs. We discuss further limitations in supplementary text section E.

Although our work focused on estimating the impact of NPIs on the reproduction number *R_t_*, the ultimate goal of governments may be to reduce the incidence, prevalence, and excess mortality of COVID-19. For this, controlling *R_t_* is essential, but the contribution of NPIs toward these goals may also be mediated by other factors, such as their duration and timing (*32*), periodicity and adherence (*33*, *34*), and successful containment (*35*). While each of these factors addresses transmission within individual countries, it can be crucial to also synchronize NPIs between countries, given that cases can be imported (*36*).

Many governments around the world seek to keep *R_t_* below 1 while minimizing the social and economic costs of their interventions. Our work offers insights into which areas of public life are most in need of virus containment measures so that activities can continue as the pandemic develops; however, our estimates should not be taken as the final word on NPI effectiveness.

## Materials and methods

### Dataset

We analyzed the effects of NPIs (Table 1) in 41 countries (*37*) (Fig. 1). We recorded NPI implementations when the measures were implemented nationally or in most regions of a country (affecting at least three-fourths of the population). We recorded only mandatory restrictions, not recommendations. Supplementary text section G details how edge cases in the data collection were handled. For each country, the window of analysis starts on 22 January and ends either after the first lifting of an NPI or on 30 May 2020, whichever came first. The reason to end the analysis after the first major reopening (*38*) was to avoid a distribution shift. For example, when schools reopened, it was often with safety measures, such as smaller class sizes and distancing rules. It is therefore expected that contact patterns in schools will have been different before school closure compared with after reopening. Modeling this difference explicitly is left for future work. Data on confirmed COVID-19 cases and deaths were taken from the Johns Hopkins CSSE COVID-19 Dataset (*13*). The data used in this study, including sources, are available online (*14*).

#### Data collection

We collected data on the start and end dates of NPI implementations, from the start of the pandemic until 30 May 2020. Before collecting the data, we experimented with several public NPI datasets, finding that they were not complete enough for our modeling and contained incorrect dates (*39*). By focusing on a smaller set of countries and NPIs than these datasets, we were able to enforce strong quality controls: We used independent double entry and manually compared our data with public datasets for cross-checking.

First, two authors independently researched each country and entered the NPI data into separate spreadsheets. The researchers manually researched the dates using internet searches: There was no automatic component in the data-gathering process. The average time spent researching each country was 1.5 hours per researcher. Next, the researchers independently compared their entries against two public datasets, the Epidemic Forecasting Global NPI (EFGNPI) Database (*40*) and the Oxford COVID-19 Government Response Tracker (*41*), and, if there were conflicts, visited all primary sources to resolve the conflicts. After that, each country and NPI was again independently entered by one to three paid contractors, who were provided with a detailed description of the NPIs and asked to include primary sources with their data. A researcher then resolved any conflicts between this data and one (but not both) of the spreadsheets. Finally, the two independent spreadsheets were combined and all conflicts resolved by a researcher. The final dataset contains primary sources (government websites and/or media articles) for each entry.

#### Data preprocessing

When the case count is small, a large fraction of cases may be imported from other countries and the testing regime may change rapidly. To prevent this from biasing our model, we neglected case numbers before a country had reached 100 confirmed cases and fatality numbers before a country had reached 10 deaths. We included these thresholds in our sensitivity analysis (fig. S13).

### Brief model description

**Fig. 5:**
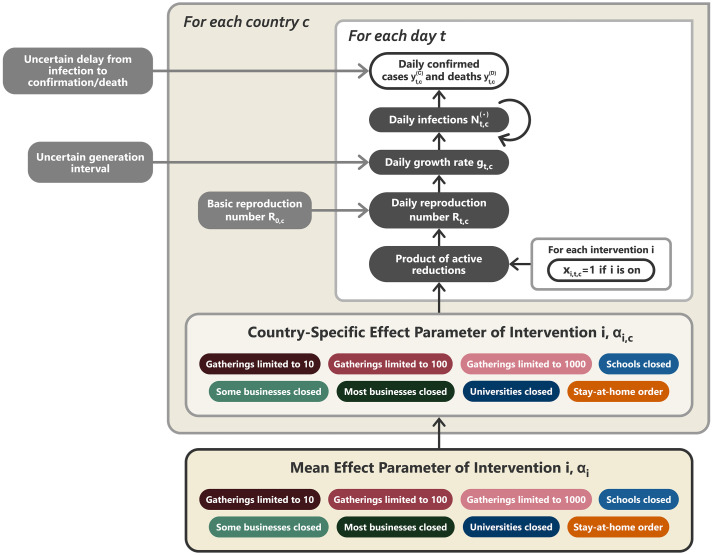
Model overview. Unshaded, white nodes are observed. From bottom to top: The mean effect parameter of NPI *i* is α*_i_*, and the country-specific effect parameter is α*_i_*_,_*_c_*. On each day *t*, a country’s daily reproduction number *R_t_*_,_*_c_* depends on the country’s basic reproduction number *R*_0,_*_c_* and the active NPIs. The active NPIs are encoded by *x_i,t,c_*, which is 1 if NPI *i* is active in country *c* at time *t*, and 0 otherwise. *R_t_*_,_*_c_* is transformed into the daily growth rate *g_t_*_,_*_c_* using the generation interval parameters and subsequently is used to compute the new infections $N_{t,c}^{\left(C\right)}$ and $N_{t,c}^{\left(D\right)}$ that will subsequently become confirmed cases and deaths, respectively. Finally, the expected numbers of daily confirmed cases $y_{t,c}^{\left(C\right)}$ and deaths $y_{t,c}^{\left(D\right)}$ are computed using discrete convolutions of $N_{t,c}^{\left(.\right)}$ with the relevant delay distributions. Our model uses both case and death data; it splits all nodes above the daily growth rate *g_t_*_,_*_c_* into separate branches for deaths and confirmed cases. We account for uncertainty in the generation interval, infection-to–case confirmation delay, and the infection-to-death delay by placing priors over the parameters of these distributions.

In this section, we give a short summary of the model (Fig. 5). The detailed model description is given in supplementary text section A. Briefly, our model uses case and death data from each country to “backward” infer the number of new infections at each point in time, which is itself used to infer the reproduction numbers. NPI effects are then estimated by relating the daily reproduction numbers to the active NPIs, across all days and countries. This relatively simple, data-driven approach allowed us to sidestep assumptions about contact patterns and intensity, infectiousness of different age groups, and so forth that are typically required in modeling studies. Code is available online (*14*).

Our model builds on the semimechanistic Bayesian hierarchical model of Flaxman *et al*. (*1*), with several additions. First, we allow our model to observe both case and death data. This increases the amount of data from which we can extract NPI effects, reduces distinct biases in case and death reporting, and reduces the bias from including only countries with many deaths. Second, since epidemiological parameters are only known with uncertainty, we place priors over them, following recent recommended practice (*42*). Third, as we do not aim to infer the total number of COVID-19 infections, we can avoid assuming a specific infection fatality rate (IFR) or ascertainment rate (rate of testing). Fourth, we allow the effects of all NPIs to vary across countries, reflecting differences in NPI implementation and adherence.

We now describe the model by going through Fig. 5 from bottom to top. The growth of the epidemic is determined by the time- and country-specific reproduction number *R_t_*_,_*_c_*, which depends on (i) the (unobserved) basic reproduction number in country *c*, *R*_0,_*_c_*, and (ii) the active NPIs at time *t*. *R*_0,_*_c_* accounts for all time-invariant factors that affect transmission in country *c*, such as differences in demographics, population density, culture, and health systems (*43*).

Following Flaxman *et al*. and others (*1*, *6*, *8*), each NPI is assumed to independently affect *R_t_*_,_*_c_* as a multiplicative factor

$$R_{t,c}=R_{0,c}\prod_{i=1}^{I}\exp\left(-\alpha_{i,c}x_{i,t,c}\right)$$

where *x_i,t,c_* = 1 indicates that NPI *i* is active in country *c* on day *t* (*x_i,t,c_* = 0 otherwise), *I* is the number of NPIs, and α*_i_*_,_*_c_* is the effect parameter for NPI *i* in country *c*. The multiplicative effect encodes the plausible assumption that NPIs have a smaller absolute effect when *R_t_*_,_*_c_* is already low.

We assume that the effect of each NPI on *R_t_*_,_*_c_* is stable across time but can vary across countries to some degree. Concretely, the effect parameter of intervention *i* in country *c* is defined as α*_i_*_,_*_c_* = α*_i_* + z*_i_*_,_*_c_*, where α*_i_* represents the mean effect parameter, and $z_{i,c}\sim N(0,\sigma_{i}^{2})$. The variance σ*_i_*^2^ corresponds to the degree of cross-country variation in the effectiveness of NPI *i* and is inferred from the data. This partial pooling of NPI effect parameters minimizes bias from country-specific sources while also reflecting that NPI effectiveness is likely different across countries. We define the effectiveness of NPI *i* as the percentage reduction in *R_t_* associated with NPI *i* across countries. This effectiveness, displayed in Figs. 2 to 4, is computed as 1 – exp(–(α*_i_* + *z_i_*)), where again $z_{i}\sim N(0,\sigma_{i}^{2})$ and σ*_i_*^2^ is drawn from its posterior. We place an asymmetric Laplace prior on α*_i_* that allows for both positive and negative effects but places 80% of its probability mass on positive effects, reflecting that NPIs are more likely to reduce *R_t_*_,_*_c_* than to increase it.

In the early phase of an epidemic, the number of new daily infections grows exponentially. During exponential growth, there is a one-to-one correspondence between the daily growth rate and *R_t_*_,_*_c_* (*44*). The correspondence depends on the generation interval (the time between successive infections in a chain of transmission), which we assume to have a gamma distribution. The prior on the mean generation interval has a mean of 5.06 days, derived from a meta-analysis (*45*).

We model the daily new infection count separately for confirmed cases and deaths, representing those infections that are subsequently reported and those that are subsequently fatal. However, both infection numbers are assumed to grow at the same daily rate in expectation, allowing the use of both data sources to estimate each α*_i_*. The infection numbers translate into reported confirmed cases and deaths after a delay. The delay is the sum of two independent distributions, assumed to be equal across countries: the incubation period and the delay from onset of symptoms to confirmation. We put priors over the means of both distributions, resulting in a prior over the mean infection-to-confirmation delay with a mean of 10.92 days (*45*) (see supplementary text section A.3). Similarly, the infection-to-death delay is the sum of the incubation period and the delay from onset of symptoms to death, and the prior over its mean has a mean of 21.8 days (*45*). Finally, as in related models (*1*, *6*), both the reported cases and deaths follow a negative binomial output distribution with separate inferred dispersion parameters for cases and deaths.

Using a Markov chain Monte Carlo (MCMC) sampling algorithm (*46*), this model infers posterior distributions of each NPI’s effectiveness while accounting for cross-country variations in effectiveness, reporting, and fatality rates as well as uncertainty in the generation interval and delay distributions. To analyze the extent to which modeling assumptions affect the results, our sensitivity analysis included all epidemiological parameters, prior distributions, and many of the structural assumptions introduced above. MCMC convergence statistics are shown in fig. S19.

## Supplementary Materials

science.sciencemag.org/content/371/6531/eabd9338/suppl/DC1 Supplementary Text Figs. S1 to S24 Tables S1 to S8 References (*47*–*79*) MDAR Reproducibility Checklist
